# Intestinal Metabolism of Crocin and a Pharmacokinetics and Pharmacodynamics Study in the Chronic Social Defeat Stress Mouse Model

**DOI:** 10.3390/ph17070843

**Published:** 2024-06-27

**Authors:** Fan Xiao, Yulong Song, Guangji Wang, Jiye Aa

**Affiliations:** Key Lab of Drug Metabolism & Pharmacokinetics, State Key Laboratory of Natural Medicines, China Pharmaceutical University, Tongjiaxiang 24, Nanjing 210009, China; xianfengwunian@163.com (F.X.); yulongsongnj@163.com (Y.S.); gjwang@cpu.edu.cn (G.W.)

**Keywords:** crocin, crocetin, depression, gut flora, drug metabolism

## Abstract

Orally administered crocin rapidly and efficiently rescues depressive-like behaviors in depression models; however, crocin levels in the circulatory and central nervous systems are rather low. The underlying mechanism responsible for the inconsistency between pharmacokinetics and pharmacodynamics is unknown. To identify the active metabolites and clarify the underlying mechanisms, the pharmacokinetics and metabolic effects of the gut flora and hepatic and intestinal microsomes on crocin were examined, and the pharmacodynamics of crocin and its major metabolite, crocetin, were also evaluated in both normal and pseudo germ-free mice subjected to chronic social defeat stress. The results showed that oral administration of 300 mg/kg crocin significantly improved the depression-like behaviors of chronic social defeat stress mice, although the levels of crocin in the circulatory system were rather low (C_max_ = 43.5 ± 8.6 μg/L; AUC = 151 ± 20.8 μg·h/L). However, the primary metabolite of crocetin was much more abundant in vivo (C_max_ = 4662.5 ± 586.1 μg/L; AUC = 33,451.9 ± 3323.6 μg·h/L). Orally administered crocin was primarily metabolized into crocetin by the gut flora instead of hepatic or intestinal microsomal enzymes, and less than 10% of crocin was transformed into crocetin in the liver or intestinal microsomes. Inhibition of the gut flora dramatically reduced the production of and in vivo exposure to crocetin, and the rapid antidepressant effect of crocin disappeared. Moreover, crocetin showed rapid antidepressant effects similar to those of crocin, and the effects were independent of the gut flora. In conclusion, the metabolic transformation of crocin to crocetin primarily contributes to the rapid antidepressant effects of crocin and is dependent on the gut flora.

## 1. Introduction

Major depressive disorder is a highly prevalent psychiatric disorder characterized by low mood, deprivation of pleasure, hopelessness, sleep difficulties, and even suicidal impulses in severe cases [1,2]. Currently, clinical antidepressant medications based on the monoamine hypothesis have a limited therapeutic effect, requiring 2–3 weeks to take effect, and are ineffective in approximately 1/3 of patients [3]. The emerging antidepressant drug, ketamine, has a rapid antidepressant effect; however, its wide use is limited due to its side effects and addictive properties [4]. Therefore, there is an urgent need to find safe and effective rapid antidepressant drugs.

**Figure 1:**
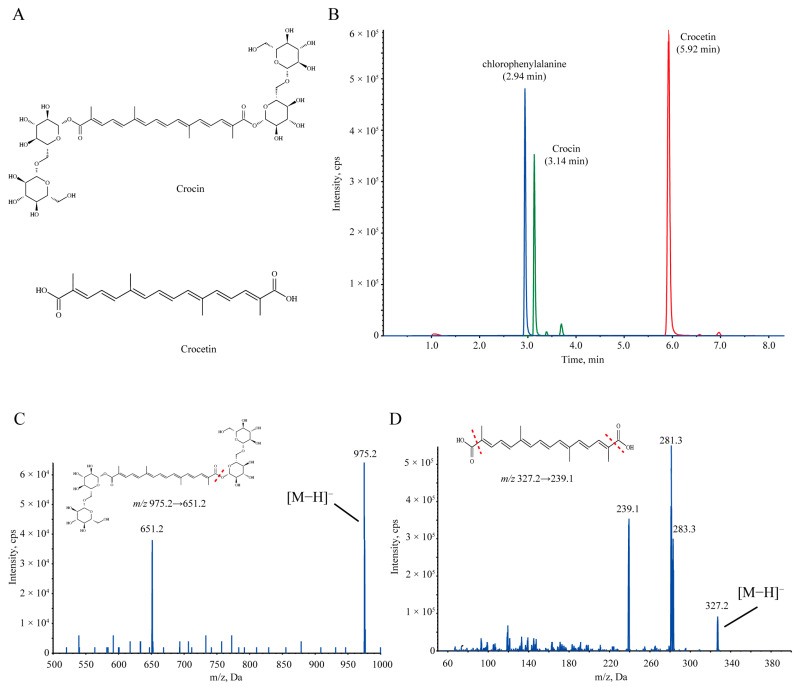
(**A**) Chemical structures of crocin (top) and crocetin (bottom). (**B**) HPLC-MS/MS chromatograms of crocin, crocetin, and chlorophenylalanine (internal standard). (**C**) The mass spectra and product ions of crocin. (**D**) The mass spectra and product ions of crocetin.

Saffron is the dried stigma of *Crocus sativus Linn. (Iridaceae)*. It is widely used as a traditional Chinese medicine for blood circulation and stasis, sedation, and hypnosis [5,6,7]. Previous studies have found that crocin (Figure 1A), the active ingredient contained in saffron, has a variety of pharmacological effects, such as anti-inflammatory [8,9], antioxidant [10,11], and neuroprotective effects [12]. In recent years, the antidepressant effects of crocin have been extensively studied. For example, multiple doses of crocin attenuated LPS-induced depressive-like behaviors by inhibiting NF-kB and NLRP3 signaling pathways and alleviated depression-like behaviors in chronic restraint stress mice by modulating the gut–brain axis [13,14]. Moreover, the antidepressant efficacy of long-term administration of crocin has not only been reported in depression model animals [14,15,16] but has also been verified in some clinical experimental studies [17,18]. In a double-blind controlled clinical trial, patients with major depression who were randomly assigned to receive either citalopram or crocin for 6 weeks showed a significant improvement in depression scores, with no significant difference noted between the two groups [19]. Furthermore, our recent data showed that, unlike previous studies on the antidepressant efficacy of long-term administration of crocin, a single high dose (300 mg/kg) of crocin rapidly and robustly reduced depressive-like behaviors in chronic social defeat stress (CSDS) models [20,21]. These findings suggest that crocin is a potential rapid antidepressant drug candidate.

Interestingly, previous pharmacokinetics (PK) studies of orally administered crocin in conventional rats have shown that the bioavailability of orally administered crocin was very low. Even when administered at high doses, the plasma concentration of crocin remained low, whereas its aglycone, crocetin, was abundant [22,23,24,25,26]. Crocetin (Figure 1A), another important component of saffron, can also be produced in vivo after oral administration of crocin [25,26,27]. Studies have demonstrated that crocetin also has an excellent antidepressant effect, and it can better cross the intestinal barrier and enter the blood circulation than crocin [22,28,29]. Therefore, there may be a significant inconsistency between PK and pharmacodynamics (PD), and this inconsistency may be related to its metabolite crocetin.

PK parameters are affected by a variety of factors, and different administration methods, dosages, and objects have great impacts on drug metabolism. Therefore, it is important to elucidate the metabolic characteristics of crocin in depressed model animals and clarify the relationship between the pharmacokinetics and pharmacodynamics of crocin and the underlying mechanism.

In the present study, we systematically explored the metabolic characteristics of orally administered crocin and used CSDS model mice to study the PK/PD of crocin in rapid antidepressant effects. As first, we conducted a detailed analysis of the pharmacokinetic profiles of crocin and its primary metabolite, crocetin, focusing on their behaviors following oral administration in a CSDS mouse model as these mice are known to exhibit depressive-like symptoms. Then, using in vivo and in vitro analyses, the location and mechanism of crocin metabolism into crocetin were systematically analyzed. Finally, we inhibited the metabolic conversion of crocin and further investigated the actual pharmacodynamic substances of crocin as a rapid antidepressant and assessed its correlation with antidepressant efficacy.

## 2. Results

### 2.1. Pharmacokinetics and Pharmacodynamics Study of Crocin in CSDS Model Mice

**Figure 2:**
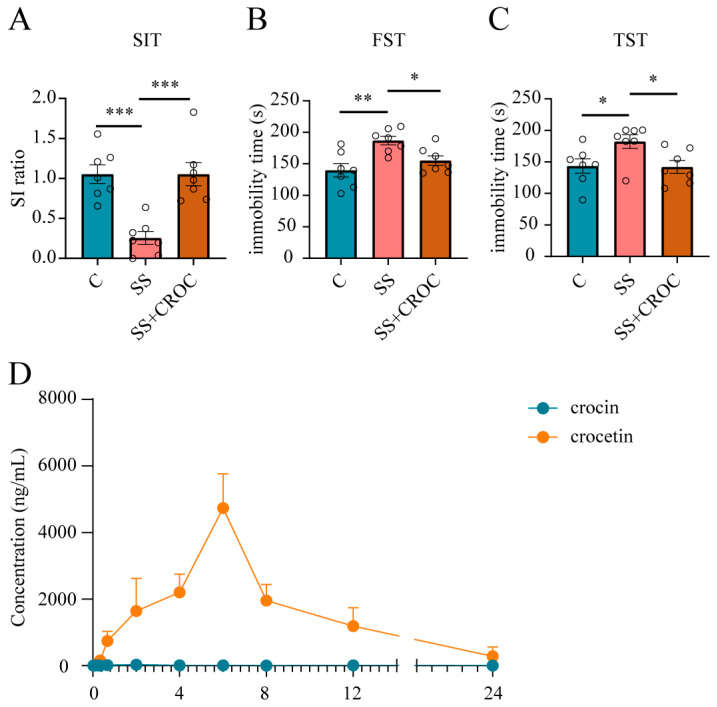
PK and PD of crocin in CSDS model mice. Effects of crocin on SIT (**A**), FST (**B**), and TST (**C**) in SS mice. The number of experimental subjects is indicated by the dots in the figures. Data are shown as mean ± SEM (*n* = 7), * *p* < 0.05, ** *p* < 0.01, *** *p* < 0.005 vs. SS group. (**D**) Mean plasma concentration–time curves of crocin and crocetin; error bars represent mean ± SEM *(n* = 3). CROC, crocin.

First, we examined the effect of the oral administration of 300 mg/kg crocin on depression-like behavior in susceptible (SS) mice. It rescued social avoidance in the social interaction test (SIT) (Figure 2A) and reduced immobilization times in the forced swimming test (FST) and tail suspension test (TST) (Figure 2B,C), showing a promising antidepressant effect. Moreover, we used a previously established analytical HPLC-MS/MS method to detect the plasma drug concentration in SS mice after crocin administration (Figure 1B–D), and the mean plasma drug concentration–time curves are shown in Figure 2D and Table 1. Surprisingly, after the administration of such a high dose of crocin, its distribution in plasma remained very limited. This low bioavailability is clearly inconsistent with the excellent antidepressant effects it has shown. We therefore speculated that there must be other physiological processes involved in regulating the bioavailability of crocin after oral administration because we also detected crocetin, the metabolite of crocin, which exhibited a much higher plasma drug concentration than crocin.

### 2.2. Metabolism of Orally Administered Crocin Both In Vivo and In Vitro

**Figure 3:**
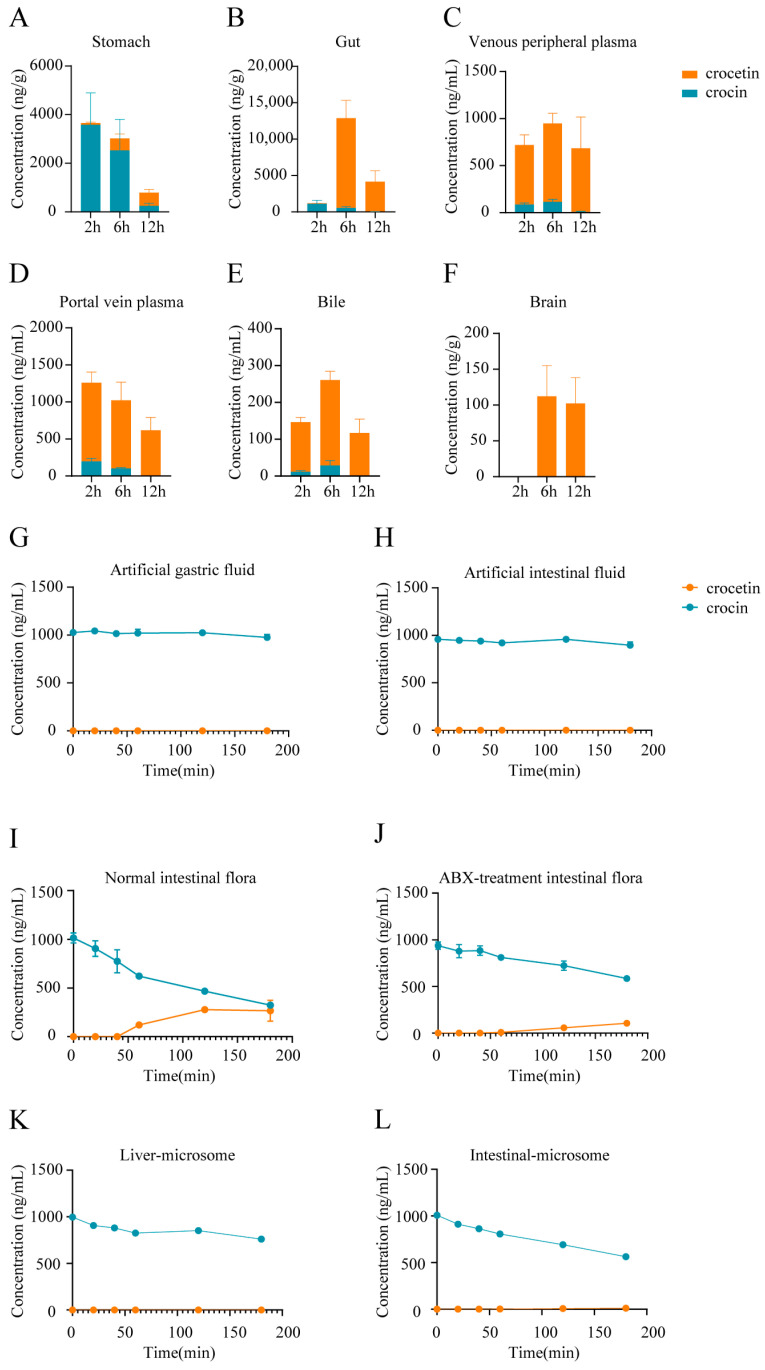
Studies on the metabolic positions of crocin in rats. Crocin and crocetin concentrations in stomach tissues (**A**), intestinal tissues (**B**), peripheral plasma (**C**), hepatic portal plasma (**D**), bile (**E**), and brain (**F**) after a single administration of crocin (100 mg/kg) over time. Error bars represent mean ± SEM (*n* = 4). The metabolism of crocin and crocetin in artificial gastric fluids (**G**), artificial intestinal fluids (**H**), fecal suspensions of C group rats (**I**), fecal suspensions of ABX group rats (**J**), liver microsomal culture medium (**K**), and intestinal microsomal culture medium (**L**). Error bars represent mean ± SEM (*n* = 3).

To further elucidate the details of the metabolism of orally administered crocin, we analyzed the concentration of crocin and the compositional ratios of crocetin in various tissues of rats after a single administration. The results showed that after oral administration of crocin, the levels of crocetin were much greater in intestinal tissues, hepatic portal vein plasma, peripheral plasma, bile, and the brain, except in gastric tissues, where crocin was detected abundantly (Figure 3A–F). This finding suggested that most crocin is first metabolized into crocetin in intestinal tissues before it enters the circulatory system.

To further investigate the factors affecting the conversion of crocin, we performed in vitro metabolism experiments using crocin. Crocin remained relatively stable in artificial gastric fluid (Figure 3G) and artificial intestinal fluid (Figure 3H), and no production of crocetin was detected. In contrast, crocin was rapidly metabolized, and crocetin was produced in the intestinal flora cultures (Figure 3I). In addition, the metabolic conversion rate of crocin was significantly reduced after the inhibition of the intestinal flora (Figure 3J). Finally, the results of microsomal in vitro metabolism experiments also showed that the contribution of hepatic and intestinal microsomes to the metabolic conversion of crocin to crocetin was also very limited (Figure 3K,L).

**Figure 4:**
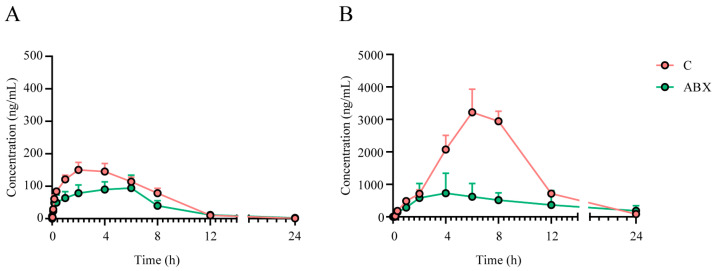
Effect of intestinal flora on the PK of crocin and crocetin after crocin oral administration. Mean serum concentration–time curve of crocin (**A**) and crocetin (**B**) following the administration of a single oral dose of crocin (100 mg/kg) to C and ABX rats. Each value represents the mean ± SEM of 12 animals.

Finally, we simultaneously evaluated the PK characteristics of crocin administered by oral gavage to rats in the gut flora inhibition group (ABX group) and the control group (C group), the parameters of which are shown in Table 2. The results showed that the plasma drug concentrations and AUCs of crocin and crocetin were significantly lower in the ABX group than in the C group, suggesting that antibiotic cocktail (ABX) treatment interferes with the bacterial flora and affects the rate of absorption of crocin (Figure 4A,B).

In conclusion, orally administered crocin is metabolized into crocetin primarily by the intestinal flora, after which it enters the circulatory system.

### 2.3. Inhibition of the Gut Flora Reduced the Production of Crocetin and Affected the Antidepressive Efficacy of Crocin

**Figure 5:**
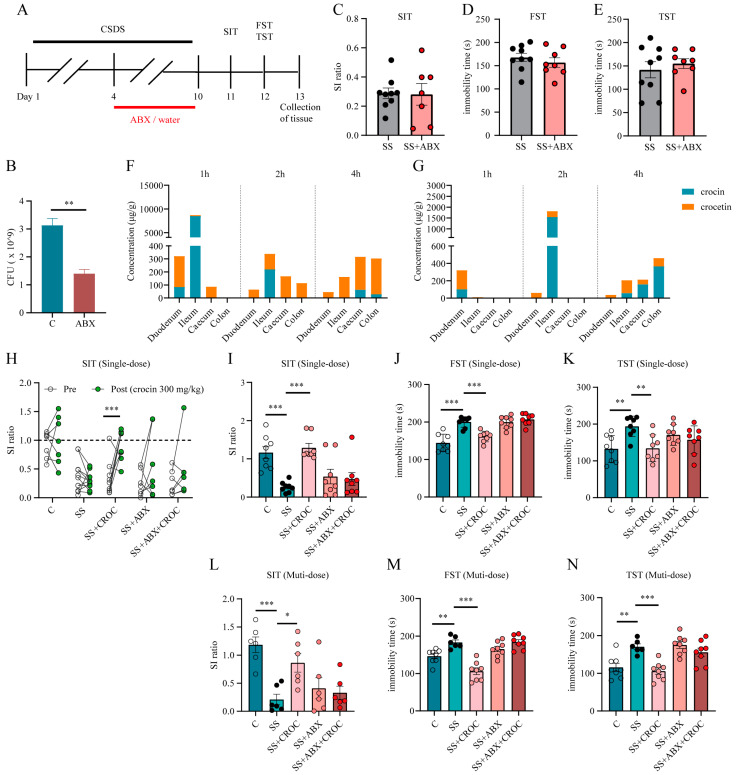
Effect of the intestinal microbiota on crocin metabolism and efficacy. (**A**) The schedule of ABX treatment, CSDS paradigm, and behavioral testing. (**B**) Number of intestinal bacteria. Data are shown as mean ± SEM (n = 3), ** *p* < 0.01. (**C**–**E**) Effects of antibiotic treatment on SIT, FST, and TST in SS mice. The number of experimental subjects is indicated by the dots in the figures. Data are shown as mean ± SEM (*n* = 7–9). Distribution of crocin and crocetin in various intestinal segments of C group (**F**) and ABX group (**G**) mice. Data are shown as mean ± SEM (*n* = 4). (**H**) Individual SI ratio values before and after the administration of a single oral dose of crocin, *** *p* < 0.001. The SI ratio value in the SIT (**I**) and the immobility times in the FST (**J**) and TST (**K**) after the administration of a single oral dose of crocin. The number of experimental subjects is indicated by the dots in the figures. Data are shown as mean ± SEM (*n* = 7–9), ** *p* < 0.01, *** *p* < 0.001 vs. SS group. The SI ratio value in the SIT (**L**) and the immobility times in the FST (**M**) and TST (**N**) after multiple doses of crocin. The number of experimental subjects is indicated by the dots in the figures. Data are shown as mean ± SEM (*n* = 6–9), * *p* < 0.05, ** *p* < 0.01, *** *p* < 0.001 vs. SS group.

To investigate the effect of crocin metabolism on antidepressant efficacy, we induced depression-like behavior in mice using the CSDS paradigm. Seven days before the end of the CSDS paradigm, half of the mice were randomly selected to be given the antibiotic mixture, and the other half were given drinking water. Each half was subsequently screened for susceptible (SS or SS + ABX) mice (Figure 5A). As a result, the number of culturable intestinal bacteria was reduced by more than half after ABX treatment (Figure 5B), suggesting that our ABX treatment effectively inhibited the flora. In addition, the behavioral results showed that there was no significant difference in the social ratio in the SIT or the immobility time in the FST and TST between the screened SS group and the SS + ABX group (Figure 5C–E). In addition, the impaired metabolism of antibiotic-treated mice was confirmed: antibiotic-treated SS mice not only had a low intestinal propulsion rate but also had a relatively lower content of crocetin in most intestinal segments (Figure 5F,G).

Then, we used metabolically impaired SS mice to study the effect of crocin metabolism on its antidepressant efficacy. As expected, crocin (300 mg/kg) ameliorated social avoidance and behavioral despair (shown as an elevated SI ratio and reduced immobility times in the FST and TST) in SS mice after a single oral administration (Figure 5H–K). However, for SS + ABX mice, the same dose of crocin failed to achieve the improvement in depressive-like behaviors that it did in the SS group (Figure 5H–K). Moreover, the SI ratio was not improved, and the immobility times in the FST and TST did not decrease in SS + ABX mice after multiple administrations (300 m/kg, 3 days) (Figure 5L–N).

This study revealed that the antidepressant effect of crocin is impaired after ABX treatment. However, regarding the cause of the loss of efficacy, is the metabolism of crocin impaired, or is other form of physiological homeostasis maintained by the intestinal flora disrupted? Further exploration is still needed.

### 2.4. Crocetin Is an Actual Pharmacodynamic Substance That Has Rapid Antidepressant Efficacy

**Figure 6:**
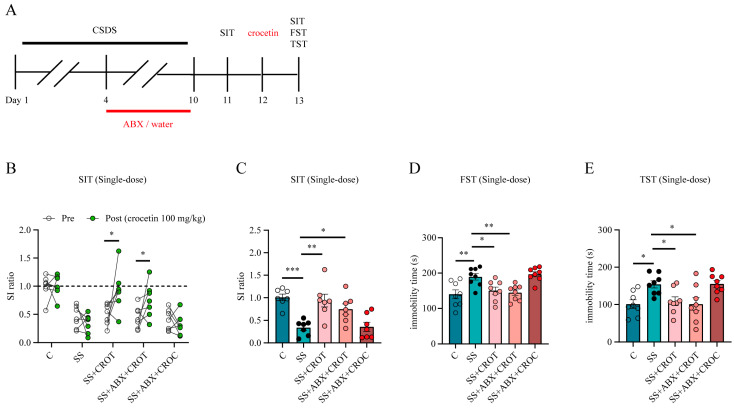
The rapid antidepressant effect of crocetin and the effect of the intestinal flora. (**A**) The experimental design of the CSDS paradigm, ABX treatment and administration of crocetin. (**B**) Individual SI ratio values before and after the oral administration of a single dose of crocetin, * *p* < 0.05. The SI ratio value in the SIT (**C**) and the immobility times in the FST (**D**) and TST (**E**) after the oral administration of a single dose of crocetin. The number of experimental subjects is indicated by the dots in the figures. Data are shown as mean ± SEM (*n* = 7–9), * *p* < 0.05, ** *p* < 0.01, *** *p* < 0.005 vs. SS group. CROT, crocetin.

As crocin exerts rapid antidepressant effects, its distribution in various tissues is mostly in the form of crocetin, and inhibiting the intestinal flora not only affects the efficiency of crocin metabolism into crocetin but also weakens the rapid antidepressant effect of crocin. Therefore, we hypothesized that the antidepressant effect of crocin was difficult to reproduce in the SS + ABX group, possibly due to the decrease in crocetin caused by the inhibition of the intestinal flora. Therefore, we investigated the therapeutic effects of crocetin on impairment of social preference and behavioral despair in SS mice, as well as the effect of ABX treatment on these parameters (Figure 6A). Compared with the SS group, a single dose of equal molar crocetin (100 mg/kg) administered by oral gavage significantly increased the SI ratio of the SS group (Figure 6B,C) and reduced the immobility times in the FST and TST (Figure 6D,E). In addition, unlike crocin, ABX pretreatment did not affect the rapid antidepressant effect of crocetin in the SS + ABX group (it elevated the SI ratio and shortened the immobility time in the FST and TST) (Figure 6B–E).

In conclusion, these data support that the key to the rapid antidepressant effect of orally administered crocin is its metabolite, crocetin.

## 3. Discussion

In recent years, the antidepressant efficacy of crocin has been extensively studied, and its antidepressant effect has been well documented [15,16,30]. For example, crocin was found to exert antidepressant effects by activating Wnt/β-catenin signaling and promoting the proliferation and differentiation of hippocampal neurons [30]. Moreover, crocin improved the synaptic plasticity of mPFC by activating mTOR and then ameliorated depressive-like behaviors in Parkinson’s disease mice [31]. However, these previous studies were based on chronic administration studies of relatively low doses of crocin. Our recent study revealed that a single oral dose of crocin was able to exert a rapid antidepressant effect by elevating cAMP in D1-MSNs in the nucleus accumbens within 24 h. It was only effective when administered at high doses, indicating its dose-dependent antidepressant efficacy [20,21]. Therefore, we studied the PK of crocin at pharmacodynamic doses in CSDS model mice and found that the results were consistent with previous PK studies in conventional rats [23,25,27]. The exposure level of crocin in vivo after administration by gavage was very low. The exposure level of crocetin, the main metabolite of crocin in the body, was significantly greater than that of crocin in the circulatory system and intestine, but it was still difficult to detect in the central nervous system.

To elucidate the PD basis of crocin and the PK factors affecting its efficacy, we conducted an in-depth study on the metabolism and PK of crocin in CSDS model mice and found that the key to the conversion of crocin to crocetin lies in the microflora rather than metabolic enzymes. Although crocin can be metabolized to crocetin in small amounts in microsomes, when we added antibiotics to inhibit the intestinal flora, the production of crocetin in the intestine was greatly reduced, the level of exposure in the body decreased, and the effect of the drug disappeared. That is, when we preserved the activity of drug-metabolizing enzymes in the gut and liver, and only the intestinal microbiota was affected, it was enough to influence the conversion of crocin to crocetin. In vitro studies have also shown that liver and intestinal microsomes are rarely involved in this metabolism. These findings suggest that the metabolism of crocin to crocetin depends on the gut microbiota rather than on liver and intestinal drug-metabolizing enzymes. Furthermore, when we tested and compared the levels of crocin and crocetin in the hepatic portal vein (intestinal absorption of drugs or metabolites that reflect the metabolism of drugs in the intestine), peripheral blood, and bile, the results were very similar. Specifically, crocin was mainly metabolized in the intestine, and this metabolism was dominant. These results suggest that crocin is mainly metabolized and converted by the intestinal flora.

The vast majority of drugs pass through the gut after oral administration, and the gut contains a large number of microorganisms that encode enzymes that can directly and significantly affect intestinal and systemic drug metabolism in mice [32,33]. These microorganisms themselves affect host health and the course of disease [34,35]. Our evidence suggests that both the gut microbiota and crocetin are closely related to the rapid antidepressant effect of oral crocin, but it is unclear which of these two factors is the independent determinant. Therefore, we further studied the influence of independent factors and found that crocin can be metabolized into crocetin through intestinal flora after oral administration. When the intestinal flora is inhibited, the conversion of crocin to crocetin is greatly reduced, and the antidepressant efficacy disappeared at the same time. Together, these data suggest that crocetin is the active molecule that exerts rapid antidepressant effects. Further tests confirmed that crocetin has a similar effect to that of crocin after administration, and the effect of crocetin was not affected after inhibiting the intestinal flora. These findings suggest that crocetin is a direct and fundamental substance for the rapid antidepressant efficacy of oral crocin and that its activity is independent of the intestinal microbiota.

## 4. Materials and Methods

### 4.1. Materials

Crocin (PubChem CID: 5281233, purity: 99.33%) and crocetin (PubChem CID: 5281232, purity: 96.61%) were purchased from Chengdu Biopurify Phytochemicals, Ltd. (Chengdu, China). The acetonitrile and methanol used were of HPLC grade and were purchased from Merck (Darmstadt, Germany). Formic acid (LC-MS grade) was purchased from Thermo Scientific (Bellefonte, PA, USA). Glucose-6-phosphate, glucose-6-phosphate dehydrogenase, β-nicotinamide adenine dinucleotide 2′-phosphate (NADPH), streptomycin sulfate, and chlorophenylalanine (as internal standards) were purchased from Sigma–Aldrich (St. Louis, MO, USA). Mixed rat live microsomes and mixed rat intestinal microsomes were purchased from the Research Institute for Liver Diseases (Shanghai, China) Co., Ltd. (Shanghai, China). Modified GAM broth base (mGAM), vitamin K1 solution, and hemoglobin chloride solution were purchased from Shandong Topbiol Co. (Shandong, China). Ampicillin, neomycin sulfate, and metronidazole were purchased from Beijing Solarbio Science & Technology Co., Ltd. (Beijing, China). Ultrapure water was generated using a Milli-Q system (Millipore, Bedford, MA, USA). All other reagents were of the highest quality commercially available.

### 4.2. Animals

Sprague-Dawley rats (180–220 g), male C57BL/6J mice (aged 6–7 weeks), and male CD1 aggressive mice (aged 6–8 months) were purchased from Vital River Experimental Animal Co., Ltd. (Beijing, China). All the animals were housed at 23 ± 2 °C and a relative humidity of 50 ± 15%. All animals were allowed free access to chow and water. All animal welfare and experimental protocols were strictly conducted in accordance with animal care laws and guidelines and approved by the China Pharmaceutical University Animal Care and Use Committee (protocol code 2021-09-039).

### 4.3. CSDS Model

The CSDS model was generated as described previously [36]. Briefly, C57BL/6 J mice were exposed to attacks by aggressive CD1 mice housed on the left side of the separated cages for 10 min per day, followed by a reduction of 0.5 min per day for 10 days. Each C57BL/6 J mouse was exposed to a different CD1 mouse each day. When the daily social attack program was over, the attacked C57BL/6 J mice were placed on the right side of their respective CD1s and allowed visual, olfactory, and auditory exposure for 24 h.

### 4.4. Behavioural Testing

#### 4.4.1. Social Interaction Test (SIT)

The SIT procedure was based on a previously described method [36]. Briefly, the ratio of the total time spent in the social area in the presence of the target to the total time spent in the absence of the target was the social interaction ratio (SI ratio). Test mice with an SI ratio less than 1 were considered susceptible (SS); otherwise, they were considered resistant.

#### 4.4.2. Forced Swimming Test (FST)

The FST was performed based on a previously described method [37]. The experimental test setup was a 5 L glass cylinder (27.5 cm high, 17.8 cm diameter) filled with 20 cm of water (24 ± 1 °C). The behavioral course of each mouse inside the cylinder was recorded for 6 min, and the immobility time of the mouse was calculated for the last 4 min.

#### 4.4.3. Tail Suspension Test (TST)

The TST was performed based on a previously described method [38]. Each mouse was secured with tape approximately 2 cm from the tip of its tail and suspended from the top of the tail box so that it was 20 cm above the ground. The behavioral course of each mouse was recorded for 6 min after suspension, and the immobility time of the mouse was calculated for the last 4 min.

### 4.5. Antibiotic Cocktail Treatment

C57BL/6J male mice were given an antibiotic cocktail (ABX) according to previously published protocols with modifications [39,40]. ABX (containing 100 mg kg^−1^ neomycin sulfate, 100 mg kg^−1^ ampicillin, and 100 mg kg^−1^ metronidazole) was administered by gavage twice a day for 7 days to inhibit intestinal bacteria. For the inhibition of intestinal flora in rats, we used streptomycin sulfate and neomycin sulfate, which were also orally administered to SD rats at a dose of 100 mg/kg twice a day for 6 days [41].

### 4.6. Chemical Analysis

Determination of crocin and crocetin in biological samples and quantitative analysis of neurotransmitters in homogenates of colon contents were achieved using an HPLC-MS/MS 5500+ (AB SCIEX, Framingham, MA, USA) system. The system includes an ExionLC binary pump, autosampler, electrospray ionization source, and 5500+ triple quadrupole mass spectrometer.

For the determination of crocin and crocetin in biological samples, 50 μL of animal plasma or tissue homogenate was mixed with 200 μL of methanol solution containing chlorophenylalanine (500 ng/mL), and then the mixtures were vortexed and centrifuged. Then, 5 μL of supernatant was injected into the LC-MS/MS 5500+ (AB SCIEX, Framingham, MA, USA) system for analysis. The chromatographic separation was achieved on an Agilent ZORBAX Eclipse Plus C18 column (3.5 μm, 4.6 × 50 mm). Multiple reaction monitoring (MRM) was performed in negative mode; crocin was detected at *m*/*z* 975.2 → 651.2, crocetin at *m*/*z* 327.2 → 239.1, and the IS chlorophenylalanine at *m*/*z* 197.8 → 137 [25].

For the quantitative analysis of neurotransmitters in homogenates of colon contents, an 80% methanol-aqueous solution containing chlorophenylalanine (500 ng/mL) was used for sample pretreatment of tissue homogenates. After centrifugation at 18,000 r/min for 10 min at 4 °C, 5 μL of supernatant was injected into the HPLC-MS/MS 5500+ (AB SCIEX, Framingham, MA, USA) system for analysis. The chromatographic separation was achieved on an Amide XBridge HPLC column (3.5 μm, 4.6 × 100 mm). Multiple reaction monitoring (MRM) was performed in positive mode, and parameters for serotonin, glutamic acid, cAMP, dopamine, norepinephrine, and GABA were 177.2 → 160.2, 148.4 → 102.1, 330.1 → 136.1, 154.1 → 137.1, 170 → 152, and 104.1 → 87, respectively [42].

### 4.7. In Vitro Metabolism of Crocin

Incubation of crocin with the intestinal flora: The intestinal contents were collected from the caecum of four ABX rats and four C rats. Fresh fecal samples from the same group were collected and pooled in sterile 50 mL tubes according to methods described in the previous literature, with some modifications [26,43,44]. Saline was added to make a 3 mL/g (33% (*w*/*v*)) suspension of intestinal flora, and a 20-fold dilution of the medium was performed for in vitro fermentation. After incubation for 12 h at 37 °C in an anaerobic environment, 50 μL of crocin solution (10 μg/mL) was added to 450 μL of bacterial culture medium. After incubation in a shaking water bath for 0 min, 20 min, 40 min, 60 min, 120 min, and 180 min, the contents of crocin and crocetin were subsequently determined.

Incubation of crocin with microsomes: Crocin (1 μg/mL) was incubated with rat liver or intestinal microsomes (1.0 mg protein/mL) in phosphate buffered saline (0.1 M, pH 7.2). After 10 min of pre-incubation at 37 °C, the reaction was initiated with a NADPH regeneration system (1 mM β-nicotinamide adenine dinucleotide phosphate, 10 mM glucose-6-phosphate, 10 mM MgCl2, and 1.0 U/mL glucose-6-phosphate dehydrogenase) in a final volume of 0.5 mL and incubated in a shaking water bath at 37 °C for 0 min, 20 min, 40 min, 60 min, 120 min, and 180 min. After incubation, samples were taken for analysis [45].

Incubation of crocin with artificial intestinal fluid and artificial gastric fluid: The preparation of artificial intestinal fluid and artificial gastric fluid was described in a previous article [46]. Crocin (1 μg/mL) was incubated with these fluids at 37 °C for 0 min, 20 min, 40 min, 60 min, 120 min, and 180 min.

### 4.8. Bacterial Flora Enumeration

Saline was added to the intestinal contents collected from the caecum of the mice to obtain a suspension of intestinal flora (3 mL/g (33% (*w*/*v*)). Serial dilutions (10 to 10^9^) of the suspension of intestinal flora were made with saline; then, 100 μL of the dilutions were inoculated onto MRS agar. The plates were incubated at 37 °C for 24 h, after which the number of colonies was calculated.

### 4.9. Pharmacokinetics Study in Rats

The rats were randomly divided into C and ABX groups. As described in the “Antibiotic cocktail treatment” section, after one week of water or ABX treatment, each rat was given a 100 mg/kg dose of crocin (*n* = 12), and blood was collected from the orbital vein of each rat (approximately 200 μL) at 0, 0.03, 0.08, 0.17, 0.33, 0.67, 1, 2, 4, 6, 8, 12, and 24 h after the administration of crocin. Pharmacokinetic parameters of crocin and crocetin were estimated using noncompartmental data analysis (NCA) in WinNonlin 6.4 software (Pharsight Corporation, Mountain View, CA, USA).

### 4.10. Tissue Distribution Study in Rats

The rats were given a 100 mg/kg dose of crocin. The animals were sacrificed at 2 h, 6 h, and 12 h after administration, and the brains, stomachs, and colons were collected immediately.

In addition, for the collection of hepatic portal blood, peripheral blood, and bile, the rats were anaesthetized with isoflurane, and a midline laparotomy was performed. Then, a catheter was positioned in the hepatic portal for sampling of hepatic portal blood, and another catheter was placed in the right femoral vein for peripheral blood sampling. Finally, bile was collected through cannulation of the bile duct.

### 4.11. Statistical Analyses

The data are expressed as the mean ± SEM. Statistical significance between two groups was evaluated using analysis of variance (unpaired *t* test). Differences among multiple groups were evaluated using one-way ANOVA (Bonferroni post hoc correction). A statistically significant difference was set at *p* < 0.05.

## 5. Conclusions

The rapid antidepressant effect of oral crocin is dependent on crocetin, which is produced by the hydrolysis of crocin in the intestines. Crocetin is the actual pharmacodynamic substance of crocin that exerts a rapid antidepressant effect. Orally administered crocin is mainly metabolized in the intestines to produce crocetin, and the metabolism is mainly achieved through intestinal flora, rather than liver or intestinal microsomal enzymes.

## Figures and Tables

**Table 1:** Pharmacokinetic parameters of crocin and crocetin following oral administration of crocin (300 mg/kg) to CSDS mice (mean ± SEM, *n* = 3).

Parameters	Crocin	Crocetin
T_max_ (h)	1	6
C_max_ (μg/L)	43.5 ± 8.6	4662.5 ± 586.1
t1/2 (h)	7.8 ± 1.7	9.4 ± 5.1
AUC (μg·h/L)	151 ± 20.8	33,451.9 ± 3323.6
V_d_ (L/kg)	12,574.45 ± 2684.7	86.4 ± 34.1
CL (L/h/kg)	1244 ± 285.4	7.8 ± 1.1

T_max_, time of maximum observed plasma concentration; C_max_, maximum plasma concentration; t1/2, terminal half-life; AUC, area in plasma under the curve by time, V_d_, apparent volume of distribution; and CL, apparent total clearance from plasma.

**Table 2:** PK parameters of crocin and crocetin following the administration of a single oral dose of crocin (100 mg/kg) to control and ABX-pretreated rats (mean ± SEM, *n* = 12). * *p* < 0.05.

Parameters	100 mg/kg, i.g.			
	Crocin		Crocetin	
	Ctrl	ABX	Ctrl	ABX
T_max_ (h)	2.8 ± 0.5	3.2 ± 0.8	7.3 ± 0.4	3.4 ± 0.6 *
C_max_ (μg/L)	198.7 ± 29.4	111.9 ± 11.5 *	4441.8 ± 690.4	942.4 ± 166.6 *
t1/2 (h)	2.1 ± 0.2	3.2 ± 0.2 *	2.7 ± 0.2	10.4 ± 2.3 *
AUC (μg·h/L)	1269.6 ± 154.7	738.9 ± 57.1 *	32186.7 ± 4275.3	8292.4 ± 1066.4 *
V_d_ (L/kg)	270.8 ± 46	696.9 ± 131.2 *	13.8 ± 1.6	166.3 ± 42.5 *
CL (L/h/kg)	90.4 ± 13.7	144.8 ± 16.8 *	3.5 ± 0.4	11 ± 1.3 *

T_max_, time of maximum observed plasma concentration; C_max_, maximum plasma concentration; t1/2, terminal half-life; AUC, area in plasma under the curve by time, V_d_, apparent volume of distribution; and CL, apparent total clearance from plasma.

## Data Availability

Data are contained within the article.
